# Insights From a Survey of School Nurses: Assessing the Challenges of Constipation in Schools

**DOI:** 10.1111/josh.70074

**Published:** 2025-09-16

**Authors:** Andrew S. Chu, Eric H. Chiou

**Affiliations:** ^1^ Department of Pediatrics, Division of Pediatric Gastroenterology, Hepatology and Nutrition Baylor College of Medicine/Texas Children's Hospital Houston Texas USA

**Keywords:** bowel management, constipation, encopresis, restroom policy, school nursing

## Abstract

**Objectives:**

School nurses are key responders to students with constipation, yet their experiences are underreported. This study surveyed their observations regarding constipation, identified barriers to student restroom access, and assessed educational needs.

**Methods:**

In this descriptive, cross‐sectional survey study, an anonymous online survey was distributed to school nurses participating in a virtual education series. The survey assessed nurse demographics, frequency of encounters with constipation and encopresis, school restroom policies, barriers to restroom use, accommodation requests, and training in constipation management.

**Findings:**

Of 125 respondents, 95% encountered students with constipation at least monthly and 55% reported ≥ 6 encounters monthly. Commonly perceived barriers to restroom use included unclean bathrooms (48%) and bullying (42%). Half of schools relied on teacher discretion for restroom access; only 37% of nurses were aware their schools had formal policies. While 64% received accommodation requests at least monthly, only 38% had training in constipation management. Most nurses expressed interest in additional education.

**Implications for School Health Policy, Practice, and Equity:**

Targeted educational interventions and school‐wide policy development are necessary to support school nurses and mitigate restrictions that could exacerbate constipation in students.

**Conclusions:**

Constipation and encopresis are frequent concerns, particularly in younger grades. Environmental and policy‐related barriers may contribute to toileting difficulties. School nurses reported variable communication with providers and the need for further education.

## 1 Introduction

Constipation is a common condition involving infrequent and/or painful passage of bowel movements, affecting approximately 1.7 million children annually in the United States [1]. Globally, childhood constipation is estimated to affect up to 30% of children [1, 2]. A variety of factors can lead to constipation, with the most common form in children being functional constipation as defined by the Rome IV criteria, comprising an estimated 90% of pediatric constipation cases [3, 4]. Constipation is one of the most frequent concerns seen by pediatric healthcare providers, representing up to 5% of visits to general pediatricians and 25% of visits to pediatric gastroenterologists [5]. It also tends to be a persistent issue, with one study finding that nearly 40% of children who had been treated for constipation by primary care providers remained constipated after 2 months of treatment [6]. Children with constipation are also more likely to use health services than their healthy peers, leading to an estimated additional healthcare cost of $3.9 billion annually [1]. As constipation progresses in severity, it can lead to encopresis, which involves involuntary stool leakage due to impaired anorectal function from chronic stool retention.

As a key body function, the passage of stool is intimately intertwined with a child's life experience. The propensity to experience constipation can be influenced by a child's environment, psychosocial factors, and host factors including underlying medical conditions. Philips et al. [7] found that life events such as separation from a best friend, failing an examination, severe illness in a close family member, loss of job by a parent, and frequent punishment were associated with constipation. At the same time, a child's experience with constipation can have direct and indirect downstream effects upon a child's social well‐being and behavioral health. Children with constipation can exhibit higher levels of anxiety and depression symptoms, social difficulties, and disruptive behavior [8]. They have been found to experience more stressful life events when compared to healthy peers, such as bullying, relational victimization, interrupted toilet training, parental punishment during toilet training, and hospitalization [7, 9, 10]. Overall, children with constipation experience lower health‐related quality of life and more somatic symptoms compared to their peers [11].

An important consideration is that children spend substantial amounts of time away from home under the supervision of staff while attending school. As a result, both students and school staff face unique challenges to the prevention and management of constipation at school. For example, much of the time spent at school may be sedentary, with one meta‐analysis finding that children spend on average 63% of their time in school being sedentary [12]. This may occur along with reduced bathroom utilization and limit the beneficial effects of physical movement on bowel activity [13]. Another challenge is stool withholding, a frequent behavior exhibited by younger school‐aged children that can exacerbate constipation. Certain factors at school, such as perceived unclean toilets, inadequate access to water, and lack of privacy, have been described as potential factors that may lead to restroom avoidance and withholding behavior at schools [14, 15, 16].

With their healthcare training and close integration into schools, school nurses can act as critical advocates for children experiencing challenges with constipation and encopresis in schools. Much of the medical literature to date regarding school nurses and constipation in schools focuses on providing nurses with recommendations regarding the care of constipation [17, 18, 19, 20]. However, despite the critical importance of this community of care providers, relatively little formal study has been done to explore the experiences and needs of school nurses regarding this issue. The goal of this study was to elicit observations from school nurses regarding their experiences caring for students with constipation, obtain feedback about potential obstacles to restroom usage, and assess their educational needs utilizing an online survey.

## 2 Methods

### 2.1 Participants

This study was a descriptive, cross‐sectional survey designed to assess the experiences of school nurses managing students with constipation in school settings. A potential survey cohort was identified by our hospital's community outreach coordinator using the enrollment list for the Texas Children's Hospital Monthly Virtual School Nursing Professional Development Series, which hosts a series of online continuing education seminars for school nurses on a variety of health‐related topics throughout the year.

### 2.2 Instrumentation

A 20‐question survey was developed using Microsoft Forms to collect information from school nurses regarding their background characteristics and observations regarding their experiences working with students experiencing constipation. The questions were thematically focused on barriers to students' bathroom use, school bathroom policies, nursing collaboration with families and medical providers, and nurses' educational needs (Appendix). The survey was addressed to nurses responsible for covering pre‐school through university levels. By design, all questions required responses to be able to submit the survey unless the survey branched to follow‐up questions as specified when relevant. Multiple choice questions allowed only a single response unless multiple response availability was noted in the question.

### 2.3 Procedure

The coordinator sent a single e‐mail broadcast message to all 1000 nurses enrolled in the series in May 2024 with a link to the online Microsoft Forms survey. The survey was sent to nurses at the end of the school year to capture their cumulative experiences over the year, and to minimize email burden on participants, no reminder emails were sent. Anonymous responses were collected over a two‐week response period, after which the survey was closed. Participation in the survey was voluntary and not linked in any way to their access to the educational series, and there was no financial incentive provided. No personal data were collected, and there was no direct communication between the investigators and school nurses.

### 2.4 Data Analysis

**FIGURE 1:**
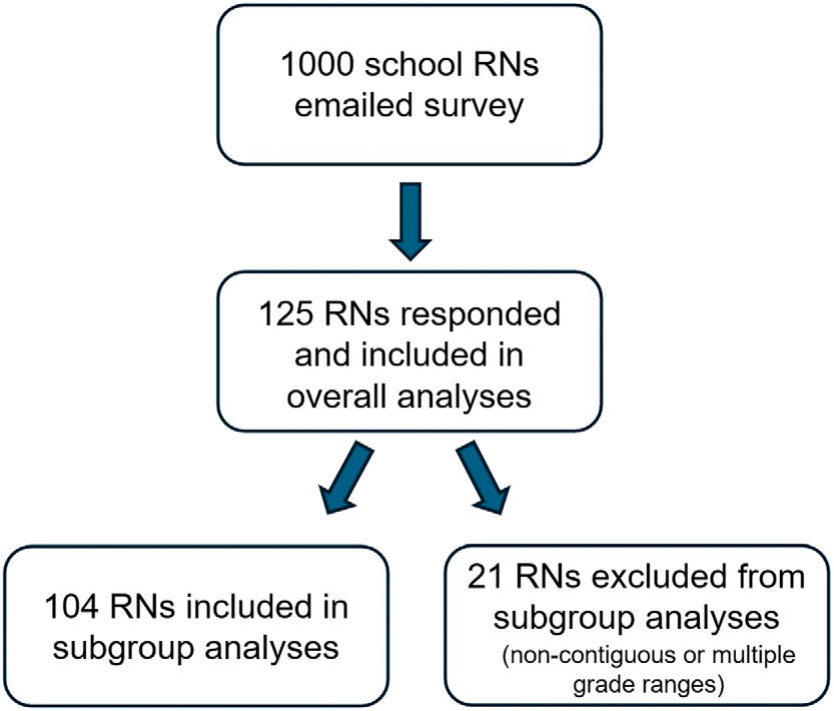
Subject enrollment.

Statistical analysis utilizing Fisher's exact test was performed using GraphPad Prism 6.0 (Boston, MA). Statistical significance was considered at *p* < 0.05. Given our sample size of 125 respondents from a population of 1000 invited nurses, the margin of error for our descriptive findings is approximately ±8.2% at a 95% confidence level, based on the standard formula Margin of Error $=z\;\times \;\sqrt{p(1-p)/n}$ with *z* = 1.96 (confidence level 95%), *n* = 125, and *p* = 0.5, which provides the most conservative estimate. For subgroup analyses comparing experiences by school levels, respondents were grouped to create developmentally coherent categories and avoid sparse data cells. Nurses covering only pre‐kindergarten and elementary grades were combined into one group (*n* = 78), while those covering only middle or high school were combined into a second group (*n* = 26). The remaining 21 nurses who covered non‐contiguous and/or more than two grade‐level ranges were excluded from these comparative analyses to maintain some degree of developmental consistency but are included in all overall descriptive statistics (Figure 1).

## 3 Results

### 3.1 Survey Response and Participant Characteristics

A total of 125 school registered nurses (RNs) responded to the survey out of 1000 invitations sent via the Texas Children's Hospital Monthly Virtual School Nursing Professional Development Series in May 2024 (Table 1). The respondents had an average of 10.9 years (range: 1–40 years) of nursing experience. Most worked in public schools, with 4% employed in private school settings. The grade levels covered by the RNs varied, with 62% of respondents serving pre‐kindergarten (Pre‐K) and elementary school students. Notably, an additional 17% of respondents had mixed responsibilities across grade levels (Pre‐K, elementary, middle and/or high school), and 79% overall covered more than one grade level.

**TABLE 1:** Characteristics of school nurse respondents.

	Mean (SD)
Years practicing as a school nurse (*n* = 125)	10.9 years (8.2)

School type (*N* = 125 total)	% (*n*/total *N*)
Public	96 (120/125)
Private	4 (5/125)

School level (*N* = 125 total)	% (*n*/total *N*)
Pre‐K and elementary school only	62 (78/125)
Middle school only	10 (12/125)
High school only	11 (14/125)
Mixed^a^	17 (21/125)
Elementary + middle school	4 (5/125)
Elementary + high school	4 (5/125)
Pre‐K through middle school	2 (3/125)
Elem through high school	1 (1/125)
Pre‐K through high school	6 (7/125)

^a^ The 21 nurses who covered non‐contiguous and/or more than two grade‐level ranges were excluded from comparative analyses to maintain developmental coherence of categories compared but are included in all overall descriptive statistics.

### 3.2 Frequency of Constipation and Encopresis Encounters in Schools

Nearly all respondents (95%) encountered students with constipation at least once per month, and over half reported encountering this more than five times a month on average (Table 2). Notably, 21% of all respondents reported seeing more than 10 students per month for constipation, while 5% reported no encounters with constipation. When analyzed by grade level, 64% of RNs covering Pre‐K and elementary school students encountered frequent cases of constipation (6 or more student cases per month) compared to 46% of middle and high school nurses (*p* = 0.11, OR 2.08, 95% CI [0.85–5.12]).

**TABLE 2:** School nurse reported average monthly frequency of stool‐related problems.

Frequency of encountering students dealing with constipation per month % (*n*/total *N*)	None	1–5	6–10	> 10	Proportion ≥ 6/months (*n*/total *N*)^a^	Comparison (pre‐K + element vs. middle + high)
Overall (*N* = 125 total overall)	5 (6/125)	40 (50/125)	34 (43/125)	21 (26/125)	55.2% (69/125)	
Pre K and elementary school (*N* = 78)^a^	6 (5/78)	29 (23/78)	37 (29/78)	27 (21/78)	64.1% (50/78)	*p* = 0.11 OR 2.08 95% CI: 0.85–5.12
Middle and high school (*N* = 26)^a^	4 (1/26)	50 (13/26)	31 (8/26)	15 (4/26)	46.2% (12/26)	
Middle school (*N* = 12)	0 (0/12)	50 (6/12)	25 (3/12)	25 (3/12)		
High school (*N* = 14)	7 (1/14)	50 (7/14)	36 (5/14)	7 (1/14)		
Other mixed levels (*N* = 21)^b^						
Elementary + middle school	0	4	1	0		
Elementary + high school	0	3	1	1		
Pre‐K through middle school	0	2	1	0		
Elem through high school	0	1	0	0		
Pre‐K through high school	0	4	3	0		

Frequency of encountering students dealing with encopresis (stool accidents) per month %(*n*/total *N*)	None	1–5	6–10	> 10	Proportion ≥ 1/month (*n*/total *N*)	Comparison (pre‐K + element vs. middle + high)
Overall (*n* = 125 total overall)	39 (49/125)	52 (65/125)	6 (8/125)	2 (3/125)	60.8% (76/125)	
Pre K and elementary school (*n* = 78)^a^	26 (20/78)	62 (48/78)	9 (7/78)	4 (3/78)	74.4% (58/78)	*p* < 0.0001 OR 7.87 95% CI: 2.88–21.5
Middle and high school (*N* = 26)^a^	73 (19/26)	27 (7/26)	0 (0/26)	0 (0/26)	26.9% (7/26)	
Middle school (*N* = 12)	75 (9/12)	25 (3/12)	0 (0/12)	0 (0/12)		
High school (*N* = 14)	71 (10/14)	29 (4/14)	0 (0/14)	0 (0/14)		
Other mixed levels (*N* = 21)^b^						
Elementary + middle school	2	3	0	0		
Elementary + high school	3	1	1	0		
Pre‐K through middle school	0	3	0	0		
Elem through high school	1	0	0	0		
Pre‐K through high school	4	3	0	0		

*Note*: Analyses were performed using GraphPad Prism 6.0. *p* values were calculated using Fisher's exact test; significance was set at *p* < 0.05. Statistical comparisons were made between the “Pre K and Elementary school” group and a combined “Middle school and High school” group.

^a^ Nurses covering only pre‐kindergarten and elementary grades were combined into one group (*n* = 78), while those covering only middle or high school were combined into a second group (*n* = 26). The 21 nurses who covered non‐contiguous and/or more than two grade‐level ranges were excluded from these comparative analyses to maintain developmental coherence of categories compared but are included in all overall descriptive statistics.

^b^ Only *N* reported without percentages due to low numbers.

Encopresis was a commonly reported issue, with 52% of RNs overall encountering one to five students per month and another 8% seeing six or more cases per month (Table 2). Collectively, middle and high school nurses were significantly less likely than Pre‐K and elementary nurses to encounter encopresis, with 73% of middle and high school nurses reporting no cases of encopresis in an average month compared with 26% of Pre‐K and elementary school nurses (*p* < 0.0001, OR 7.87, 95% CI [2.88–21.5]).

### 3.3 Barriers to Restroom Access

Most RNs (66%) reported that restroom access by students required only minimal travel from classrooms (less than a minute walk), while 24% described the distance as modest (walk for a minute or two to get to restrooms, i.e., down the hall), and 7% characterized it as moderate (may need to walk to a different part of the building and/or walk up or down a floor to get to a restroom) or significant (may need to walk out of the building to use a restroom, e.g., walking from a classroom in a portable trailer to a main school building) (Table 3). Perceived barriers to student restroom use were reported, with the most common concerns being unclean restrooms (48%), bullying (42%), drug use (23%), and vandalism (18%). Also, 42% of nurses cited additional varied concerns that included student anxiety about using school bathrooms due to lack of privacy, embarrassment (e.g., potential social media exposure), preferring their home toilet and/or not being comfortable with school restrooms, fear of the noise of flushing, fear of automatic flushing toilets, and pressure from being timed.

**TABLE 3:** School nurse observations of barriers to restroom usage and school restroom policies.

Distance from class to restroom^a^ % (*n*/total *N*)	None	Minimal	Modest	Moderate	Significant
	3 (4/125)	66 (82/125)	24 (30/125)	5 (6/125)	2 (3/125)

Frequency of restroom inspection % (*n*/total *N*)	< Once/day	Once/day	> Once/day	Don't know
	3 (4/125)	10 (12/125)	54 (67/125)	34 (42/125)

Frequency of restroom cleaning % (*n*/total *N*)	< Once/day	Once/day	> Once/day	Don't know
	2 (2/125)	31 (39/125)	38 (48/125)	29 (36/125)

Observed barriers to restroom use^b^	% (*n*/total *N*)
Broken/missing doors	18 (23/125)
Non‐working sinks/soap	12 (15/125)
Bullying	42 (53/125)
Drugs	23 (29/125)
Vandalism	18 (22/125)
Unclean bathrooms	48 (60/125)
Loose toilets	5 (6/125)
None	18 (22/125)
Other	42 (52/125)
	Lack of privacy (*n* = 13) Embarrassment and fear of social media (*n* = 12) Prefer home (*n* = 12; one preferred bidet) Fear of noise of flushing (*n* = 5) Fear of auto toilet (*n* = 2) Pressure from being timed (*n* = 1)

School approaches to privileges	% (*n*/total *N*)				
Teacher discretion	50 (62/125)				
School has policy	37 (46/125)				
RN not aware of policy	10 (13/125)				
Other	3 (4/125)				
*School RN can give pass for expanded privileges, School RN requests MD note for expanded privileges, Children can ask but restrooms are locked at certain times–go to RN in emergency*					

Management of restroom access	% (*n*/total *N*)				
Open access	51 (64/125)				
Limited by passes	29 (36/125)				
How many?	Many RNs were not certain. Generally, they indicated as‐needed access but restricted to one boy and one girl at a time. Some nurses reported a limited number of passes per day/number of weeks.				
Scheduled times	20 (25/125)				

^a^ None: restroom in the classroom. Minimal: in immediate vicinity of most classrooms (< 1 min walk). Modest: 1–2 min to restrooms (e.g., down the hall). Moderate: walk to a different part of the building and/or up or down a floor. Significant: walk out of the building to use a restroom in another building.

^b^ Respondents could select multiple options; therefore, percentages may sum to more than 100%.

In terms of restroom monitoring by schools, 54% of nurses reported that restrooms were inspected by staff more than once daily, while 34% were uncertain about restroom monitoring frequency. Regarding bathroom cleaning, 69% reported that this occurred at least once daily, and 29% of RNs were unsure about cleaning schedules.

### 3.4 School Policies on Restroom Use

Half (50%) of respondents indicated that restroom access was at teacher discretion, while 37% reported that their school had a formal policy (Table 3). Open restroom access was available in 51% of schools, 29% used a pass system, and 20% utilized scheduled restroom times. Among schools with pass systems, policies varied widely, with some restricting access to one boy and one girl at a time, while others limited the number of passes per day or per semester.

### 3.5 Communication With Parents and Medical Professionals

Parental or medical professional communication to school RNs regarding students with constipation was common, with 64% of respondents receiving at least one request per month for accommodations such as expanded restroom privileges or use of a private restroom (Table 4). Only 3% of respondents reported receiving six or more such requests per month. When students experienced a stool accident beyond the expected age of bowel control (in the RN's opinion), 90% of RNs reported notifying the child's parent and 29% informed the teacher; 7% indicated they had contacted a medical provider about the incident.

**TABLE 4:** School nurse collaboration with families/providers and educational needs.

Average monthly requests by parents and/or providers for restroom accommodations	% (*n*/total *N*)
None	37 (46/125)
1–5	61 (76/125)
6–10	2 (2/125)
> 10	1 (1/125)

RN action if child has stool accident^a^	% (*n*/total *N*)
Never encountered	7 (9/125)
Nothing	1 (1/125)
Mention to teacher	29 (36/125)
Mention to parent	90 (113/125)
Contact provider	7 (9/125)

Types of accommodation requests received over past year^a^	% (*n*/total *N*)
Increased access to restroom	82 (103/125)
Ability for student to carry water bottle	81 (101/125)
More time to use restroom	54 (67/125)
Access to private restroom	51 (64/125)
504 plan or IEP request	34 (43/125)

Has RN contacted provider about student struggling with constipation in last year?	% (*n*/total *N*)
Yes	18 (22/125)
No	82 (103/125)

Has RN received education/training on bowel problems/constipation in children^a^	% (*n*/total *N*)
Yes	38 (47/125)
*If yes, what type? (%(n) of “yes”)*	
Continuing education	62 (29/47)
Informal by providers	15 (7/47)
Personal research	17 (8/47)
Inservice	4 (2/47)
No description	2 (1/47)
No	62 (78/125)
*If no, what would be helpful? (%(n) of “no”)*	
Info on childhood constipation	83 (65/78)
Fluid intake guidelines	67 (52/78)
Info on managing toileting habits	88 (69/78)
Info on improving restrooms	44 (34/78)
Other	8 (6/78)

^a^ Respondents could select multiple options; therefore, percentages may sum to more than 100%.

### 3.6 Education and Training Needs

Most school nurses (62%) reported receiving no formal education or training on pediatric constipation management (Table 4). Among the 38% who had received training, 61% cited continuing education courses, while others reported informal learning from medical providers (15%) or personal research (17%). When asked about preferred educational resources, 88% of nurses expressed interest in learning how to identify and treat abnormal toileting habits, 83% wanted more information on childhood constipation, and 67% requested guidance on recommended fluid intake. Additional suggestions included strategies for preventing students from misusing restroom accommodations and methods for encouraging physical activity and fiber intake in schools.

### 3.7 Accommodation Requests for Students With Constipation

Over the last academic year, school nurses received a range of accommodation requests from families and medical providers (Table 4). The most common requests included increased restroom access (82%), permission for students to carry water bottles during the school day (81%), extended restroom time (54%), and access to a private restroom (51%). Requests for formal educational accommodations, such as a 504 plan or Individualized Education Program (IEP) modifications, were less frequent (34%). Overall, 18% of school nurses reported reaching out to a medical provider about a student's constipation within the last year.

## 4 Discussion

Constipation is a common and significant issue for both students and school staff, and school nurses play a key role helping to evaluate and manage the challenges that their students face from this issue. Despite their importance, little study has been conducted of school nurses and their experiences in handling pediatric constipation and encopresis. To our knowledge, the literature contains only one previous study of school nurses' perceptions of pediatric toileting with a urologic focus [21]. Our study provides unique contributions to the literature as it assesses the frequency with which school nurses encounter children at different ages with constipation and encopresis and evaluates aspects of school nurses' collaboration with parents and providers. Our study also provides the most detailed assessment to date of nurses' perceptions of school policies regarding management of restroom privileges and of their perceived barriers to students' use of school restrooms.

The findings from our survey highlight several important points. School nurses frequently deal with students experiencing constipation, with 55% of all respondents reporting six or more encounters with constipation per month. Encopresis was especially prevalent for school nurses caring for younger students, with 74% at the pre‐K/elementary level reporting one or more encounters with encopresis per month. Furthermore, a significant proportion of school nurses were responsible for a complex mix of student ages across multiple school levels. Nurses often communicate with parents regarding these concerns, with 64% of respondents reporting at least one request per month from providers and families for toileting accommodations. Direct communication between school nurses and other health care providers regarding constipation was less common. The most common barriers to restroom access were unclean bathrooms and bullying, reported by over 40% of respondents. The nurses in our study called special attention to environmental and behavioral factors that could contribute to students' anxiety about using school restrooms. School policies regarding the management of restroom privileges varied considerably, potentially suggesting a need for more structured school policies to support affected students. Finally, 62% of nurses in our study reported no formal training or education on constipation and encopresis, and of these, 83% indicated having an interest in increased education.

Pediatric constipation and encopresis significantly impact school‐aged children, influencing their physical health, emotional well‐being, and academic success [8, 22]. The reluctance of children to use school restrooms, often due to inadequate facilities, fear of bullying, and restrictive policies, exacerbates these conditions [23, 24]. Our study provides novel insights from the point of view of school nurses into the frequency of these issues in school settings and the specific barriers that impede effective management. School nurses face distinct challenges, including limited training, lack of clear policies, and insufficient coordination with medical providers [25, 26]. Previous research has demonstrated that many school nurses are unaware of best practices for managing pediatric toileting issues, which aligns with our findings that 62% had no formal training. Addressing these educational gaps through targeted professional development can enhance school nurses' ability to support affected students. Publicly available resources such as the Complete Constipation Care Package offered by the North American Society for Pediatric Gastroenterology, Hepatology & Nutrition (NASPGHAN) on its public education website GIKids.org (https://gikids.org/constipation/) can be a valuable resource for school nurses. Our study also highlights the critical role of schools in shaping children's toileting behaviors.

### 4.1 Implications for School Health Policy, Practice, and Equity

By identifying these challenges, our research provides a foundation for future interventions aimed at improving the management of pediatric constipation in school settings. Restrictive restroom policies, inadequate maintenance, and social stigma associated with encopresis contribute to withholding behaviors, which can lead to worsening constipation [27, 28]. Schools can mitigate these barriers by implementing structured bathroom policies that prioritize student needs, improving restroom cleanliness and accessibility, and fostering better communication between school staff and healthcare providers. There appears to be a clear need for further research and development of school‐directed resources that can provide evidence‐based recommendations regarding best practices for the management of constipation and restroom access in schools. This represents an opportunity for multidisciplinary collaboration among parents, patients, teachers, school administrators, school nurses, pediatricians, and pediatric specialists including gastroenterologists and urologists to develop public policies to help children and support schools in this area.

### 4.2 Limitations

Our study had several limitations. As a voluntary survey‐based study, we had little control over who responded, and so the choice of nurses to respond or not to the survey could have introduced biases into the study. For example, it is conceivable that nurses who were more interested in the topic of constipation responded at a higher rate than those who were not. The nurses who responded to this survey were almost entirely employed at public schools within a single state, so the results of this study would primarily apply to this setting; extrapolation to private school settings would therefore be limited and need to be done with caution. In addition, as the target population consisted of nurses who were enrolled in our hospital's virtual learning series, this group of nurses was essentially self‐selected for those who were motivated to pursue additional learning activities, and the use of a single e‐mail invitation without reminders may have amplified this self‐selection bias. Our subgroup analysis also excluded 21 nurses with highly varied responsibilities across grade levels, and while this improved the homogeneity of our comparison groups, their unique experiences are not captured in those specific analyses. Another limitation is that our survey was based largely on nurses' recall and their perceptions of occurrences such as how often they encountered students with constipation in a given month. It is possible that those recalls were not accurately reflective of what had occurred. A further limitation was that to prevent the length of the survey from dissuading nurses from responding, we limited the length of the survey as well as the complexity of the questions, which prevented us from pursuing a broader range of questions. Finally, our analysis was primarily descriptive. While this was appropriate for our exploratory aims, we did not perform multivariable regression analyses to adjust for potential confounders like nurse experience or school type. Future studies with larger, more diverse samples would be needed to identify the independent predictors of nurse‐reported constipation frequency and barriers.

## 5 Conclusions

This study underscores the high frequency of constipation in schools and highlights some of the barriers to restroom use that may exacerbate children's constipation. It also reveals opportunities for strengthening collaboration among school nurses, families, and medical professionals as well as bolstering the education of school nurses on this issue. An important need exists for medical professionals to work with schools and families to develop and advocate for best practices about school policies and facilities to help mitigate constipation at schools. Further studies are needed to more accurately understand the specific issues that may contribute to worsened constipation in schools and to define the best practices for schools to implement. In addition, while the prevalence of specific barriers to restroom use such as bullying or unclean restrooms may vary by region or country depending on funding and cultural factors, the fundamental challenges that nurses face such as inadequate training and the need for clear and supportive restroom policies are likely common across many school systems. Future multi‐state or international studies would be essential to confirm these findings in different contexts.

## Ethics Statement

The investigational protocol for this survey study was approved by the Institutional Review Board at Baylor College of Medicine (Houston, Texas) under protocol H‐55422.

## Conflicts of Interest

The authors declare no conflicts of interest. No honorarium, grant, or other payment was provided to anyone for production of this manuscript.

## Supporting information


**Data S1:** Supporting Information.

## Data Availability

The data that support the findings of this study are available from the corresponding author upon reasonable request.
