# Supplementary material for: Insights From a Survey of School Nurses: Assessing the Challenges of Constipation in Schools
Source: J Sch Health. 2025 Sep 16;95(12):1082–90. doi: 10.1111/josh.70074 (PMC12621158; doi:10.1111/josh.70074)
Supplement: Supplementary file 1 — Data S1: Supporting Information. [file JOSH-95-1082-s001.pdf]

Appendix. Survey Instrument

(All questions required responses to be able to submit the survey unless the survey branched as specified when relevant. Multiple choice questions allowed only a single response unless multiple response availability was noted in the question.)

1. How many years have you worked in school nursing? (enter number)
2. Which of the following school levels best describes your primary work environment? (We realize that some schools will not fit exactly into these categories. Pick the option(s) that most closely match the grades you are currently responsible for.)
    - Preschool (Pre-K)
    - Elementary (K-5)
    - Middle School (6-8)
    - High School (9-12)
    - College/University
3. Do you work at a public or private school?
    - Public
    - Private
4. In an average month, approximately how often do you encounter students dealing with constipation?
    - None per month
    - 1-5 times per month
    - 6-10 times per month
    - 10+ times per month
5. In an average month, approximately how often do you encounter students dealing with encopresis (stool leakage/accidents due to constipation)?
    - None per month
    - 1-5 times per month
    - 6-10 times per month
    - 10+ times per month
6. In an average month, approximately how often do you receive communication from parents and/or medical professionals requesting assistance for students with constipation (such as requests for expanded restroom privileges, use of private restroom, etc.)?
    - None per month
    - 1-5 times per month
    - 6-10 times per month
    - 10+ times per month
7. What do you do if a child has a stool/bowel accident after the age at which they should have achieved control? (Check all that apply for you.)
    - I have never encountered this (Note: if you select this option, do not select other options)
    - Nothing, since they will likely outgrow it (Note: If you select this option, do not select other options)
    - Mention it to the child’s teacher
    - Mention it to the child’s parent/guardian
    - Contact the child’s primary health care provider

- Other (type here)

8. How far do students at your school typically have to travel to get from a classroom to a restroom? (We realize that students at some schools may have a variety of distances they may need to travel to get to the restroom during the day. If that is the case, please select the most significant distance that a typical student may need to travel to get to a restroom.)
   - None: the restrooms are located in the classrooms
   - Minimal: restrooms are in the immediate vicinity of most classrooms (less than a minute walk).
   - Modest: students may need to walk a minute or two to get to restrooms (e.g. down the hall)
   - Moderate: students may need to walk to a different part of the building and/or walk up or down a floor to get to a restroom.
   - Significant: students may need to walk out of the building to use a restroom in another building (e.g. students walking from a classroom in a portable trailer to a main school building).
9. Medical providers sometimes hear from patients that they do not want to use school restrooms for a variety of reasons that may cause them to wait until they get home to use the restroom. Based on your personal observations as well as reports from students, faculty, and/or parents, which of the following potential barriers to student restroom use occur at your school during the year? (Check all that apply; remember your responses are confidential.)
   - Broken or missing stall doors
   - Non-working sinks and/or soap dispensers
   - Bullying in the restrooms
   - Drug use by students in the restrooms
   - Vandalism (e.g. breakage of toilets and/or urinals, extensive graffiti, etc.)
   - Unclean (e.g. urination and/or defecation on the floors, etc.)
   - Loose toilet seats
   - None of the above
   - Other (free text)
10. How often are restrooms inspected for issues by staff during the day?
    - Less than once daily
    - Once daily
    - More than once daily
    - Don’t know
11. How often are restrooms cleaned?
    - Less than once daily
    - Once daily
    - More than once daily
    - Don’t know
12. A topic that medical providers encounter in visits with children who have constipation is the management of restroom privileges at school. Which of the following best describes your school’s approach?
    - Teachers have the discretion to manage students’ access to restrooms as they see fit.
    - Our school has a general policy for students’ access to restrooms that teachers follow.

- I am not aware that our school has any policy regarding access to bathrooms.
- Other

13. Which of the following best describes the most common method for managing restroom access at your school?
    - Open access (students can freely go to the restroom when needed) *[→ if selected, survey branches to question 16)]*
    - Limited by passes (students can go if they have a pass that they can turn in that allows them to go to the restroom) *[→ if selected, survey branches to question 14)]*
    - Scheduled times (students must go during designated times) *[→ if selected, survey branches to question 15)]*
14. If you checked "Limited by passes" above, how many passes are they provided and over what time period? For example, responses might say "3 per day" or "5 per quarter". (Type answer below)
15. If you checked "Scheduled times" above, when are the most common scheduled times? For example, responses might say "after lunch" or "before recess". (Type answer below)
16. Have you received education or training on bowel problems such as constipation in children?
    - Yes *[→ if selected, branches to question 17)]*
    - No *[→ if selected, branches to question 18)]*
17. If you answered "Yes" to the last question, please describe below. (free text)
18. If you answered "No" to the last question, what resources do you think would be helpful? (check all that apply)
    - Information about childhood constipation
    - Fluid intake guidelines
    - Information about identifying and treating abnormal toileting habits
    - Information about improving restroom facilities
    - Other
19. Over the last year, has your office received requests for any of the following accommodations from families or medical providers? (check all that apply)
    - Increased access to restrooms
    - Access to a more private restroom
    - More time to use the restroom
    - Ability for student to carry a water bottle with them during the day
    - Request or recommendation to develop a 504 plan or IEP to address constipation care needs
20. Have you had to reach out to a medical provider regarding a student struggling with constipation in the last year?
    - Yes
    - No

Question	Coding
1. Years in school nursing	Continuous: (Numeric value as entered)
2. RN School level (check all that apply)	Binary (0/1) for each level:<br>0 = Not Checked<br>1 = Checked
3. Public or private school	Categorical:<br>1 = Public<br>2 = Private
4. Frequency of constipation encounters	Ordinal:<br>0 = None per month<br>1 = 1-5 times per month<br>2 = 6-10 times per month<br>3 = 10+ times per month
5. Frequency of encopresis encounters	Ordinal:<br>0 = None per month<br>1 = 1-5 times per month<br>2 = 6-10 times per month<br>3 = 10+ times per month
6. Frequency of parent/provider communication	Ordinal:<br>0 = None per month<br>1 = 1-5 times per month<br>2 = 6-10 times per month<br>3 = 10+ times per month
7. Action if child has stool accident (check all that apply)	Binary (0/1) for each action:<br>0 = Not Checked<br>1 = Checked<br>Text: (Free-text response for "Other")
8. Distance from classroom to restroom	Ordinal:<br>0 = None<br>1 = Minimal<br>2 = Modest<br>3 = Moderate<br>4 = Significant
9. Barriers to student restroom use (check all that apply)	Binary (0/1) for each barrier:<br>0 = Not Checked<br>1 = Checked<br>Text: (Free-text response for "Other")
10. Frequency of restroom inspection	Ordinal:<br>1 = Less than once daily<br>2 = Once daily<br>3 = More than once daily<br>4 = Don't know
11. Frequency of restroom cleaning	Ordinal:<br>1 = Less than once daily<br>2 = Once daily

	3 = More than once daily<br>4 = Don't know
12. School's approach to restroom privileges	Categorical:<br>1 = Teacher discretion<br>2 = School has a general policy<br>3 = Not aware of a policy<br>4 = Other
13. Method for managing restroom access	Categorical:<br>1 = Open access (if selected, survey branches to question 16)<br>2 = Limited by passes (if selected, survey branches to question 14)<br>3 = Scheduled times (if selected, survey branches to Question 15)
14. Details on pass limits	Text: (Free-text response)
15. Details on scheduled times	Text: (Free-text response)
16. Received education or training	Binary (0/1):<br>0 = No (if selected, branches to question 18)<br>1 = Yes (if selected, branches to question 17)
17. Description of training received	Text: (Free-text response)
18. Helpful resources if no training (check all that apply)	Binary (0/1) for each resource:<br>0 = Not Checked<br>1 = Checked
19. Accommodation requests received (check all that apply)	Binary (0/1) for each accommodation:<br>0 = Not Checked<br>1 = Checked
20. Reached out to a medical provider	Binary (0/1):<br>0 = No<br>1 = Yes
